# Supplementary material for: Assessing the Efficacy of the Spectrum-Aided Vision Enhancer (SAVE) to Detect Acral Lentiginous Melanoma, Melanoma In Situ, Nodular Melanoma, and Superficial Spreading Melanoma
Source: Diagnostics (Basel). 2024 Aug 1;14(15):1672. doi: 10.3390/diagnostics14151672 (PMC11312294; doi:10.3390/diagnostics14151672)
Supplement: Supplementary file 1 [file diagnostics-14-01672-s001.zip › diagnostics-3046023-supplementary.pdf]

diagnostics

MDPI

Supplementary Materials

Assessing the Efficacy of the Spectrum-Aided Vision Enhancer (SAVE) to Detect Acral Lentiginous Melanoma, Melanoma In Situ, Nodular Melanoma, and Superficial Spreading Melanoma

Teng-Li Lin [1], Chun-Te Lu [2,3], Riya Karmakar [4], Kalpana Nampalley [4], Arvind Mukundan [4], Yu-Ping Hsiao [5,6], Shang-Chin Hsieh [7,*] and Hsiang-Chen Wang [4,8,*]

1 Department of Dermatology, Dalin Tzu Chi General Hospital, No. 2, Min-Sheng Rd., Dalin Town, Chiayi 62247, Taiwan
2 Institute of Medicine, School of Medicine, College of Medicine, National Yang Ming Chiao Tung University, No. 155, Sec. 2, Li-Nong Street, Beitou District, Taipei 112304, Taiwan
3 Department of Surgery, Division of Plastic and Reconstructive Surgery, Taichung Veterans General Hospital, 1650 Taiwan Boulevard Sect. 4, Taichung 407219, Taiwan
4 Department of Mechanical Engineering, National Chung Cheng University, 168, University Rd., Min Hsiung, Chia Yi 62102, Taiwan
5 Department of Dermatology, Chung Shan Medical University Hospital, No. 110, Sec. 1, Jianguo N. Rd., South Dist., Taichung City 40201, Taiwan
6 Institute of Medicine, School of Medicine, Chung Shan Medical University, No. 110, Sec. 1, Jianguo N. Rd., South Dist., Taichung City 40201, Taiwan
7 Department of Surgery, Division of General Surgery, Kaohsiung Armed Forces General Hospital, 2, Zhongzheng 1st. Rd., Lingya District, Kaohsiung 80284, Taiwan
8 Department of Technology Development, Hitspectra Intelligent Technology Co., Ltd., Kaohsiung 80661, Taiwan
* Correspondence: sschin522@gmail.com (S.-C.H.); hcwang@ccu.edu.tw (H.-C.W.)

Citation: Lin, T.-L.; Lu, C.-T.; Karmakar, R.; Nampalley, K.; Mukundan, A.; Hsiao, Y.-P.; Hsieh, S.-C.; Wang, H.-C. Assessing the Efficacy of the Spectrum-Aided Vision Enhancer (SAVE) to Detect Acral Lentiginous Melanoma, Melanoma In Situ, Nodular Melanoma, and Superficial Spreading Melanoma. *Diagnostics* **2024**, *14*, 1672. https://doi.org/10.3390/diagnostics14151672

Academic Editor: Naresh Kumar Ravichandran

Received: 22 May 2024
Revised: 18 July 2024
Accepted: 26 July 2024
Published: 1 August 2024

Copyright: © 2024 by the authors. Submitted for possible open access publication under the terms and conditions of the Creative Commons Attribution (CC BY) license (https://creativecommons.org/licenses/by/4.0/).

Abstract: This article contains the supplementary file for the article, "Assessing the Efficacy of Spectrum Aided Visual Enhancer (SAVE) in Skin Cancer Detection using Band Selection and Hyperspectral Imaging". The first section explains about the equations used to develop the SAVE algorithm in this study. The second section gives the training curves, loss plots and the F1-confidence curve used in the study while the third section provides the confusion matrix in terms of percentage as well in terms of absolute numbers. The last section provides more examples of the detection of images of skin cancer in both RGB imaging as well as using the SAVE model.

Keywords: Skin cancer, Acral Lentiginous Melanoma, Melanoma in Situ, Nodular Melanoma, Superficial Spreading melanoma, Hyperspectral imaging, band selection, spectrum-aided visual enhancer.

1. SAVE

The visible hyperspectral imaging (VIS-HSI) used in this study is calculated by using the images taken by a single-lens camera combined with the visible hyperspectral algorithm. The wavelength range is from 380 nm to 780 nm, and the spectral resolution is up to 1 nm.

The individual conversion formulas to convert the 24-colour patch image and 24 colour patch reflectance spectrum data to XYZ colour space are as follows

On the camera side: convert sRGB color gamut space to XYZ color gamut space

$$\begin{bmatrix} X \\ Y \\ Z \end{bmatrix} = [M_A][T]\begin{bmatrix} f(R_{sRGB}) \\ f(G_{sRGB}) \\ f(B_{sRGB}) \end{bmatrix} \times 100\ , 0 \leq \begin{matrix} R_{sRGB} \\ G_{sRGB} \\ B_{sRGB} \end{matrix} \leq 1 \tag{S1}$$

$$[T] = \begin{bmatrix} 0.4104\ 0.3576\ 0.1805 \\ 0.2126\ 0.7152\ 0.0722 \\ 0.0193\ 0.1192\ 0.9505 \end{bmatrix} \tag{S2}$$

$$f(n) = \begin{cases} (\frac{n+0.055}{1.055})^{2.4}, n > 0.04045 \\ \left(\frac{n}{12.92}\right), otherwise \end{cases} \tag{S3}$$

$$[M_A] = \begin{bmatrix} X_{SW}/X_{CW} & 0 & 0 \\ 0 & Y_{SW}/Y_{CW} & 0 \\ 0 & 0 & Z_{SW}/Z_{CW} \end{bmatrix} \tag{S4}$$

On the spectrometer side: convert reflection spectral data to XYZ color gamut space

$$X = k\int_{380nm}^{780nm} S(\lambda)R(\lambda)\bar{x}(\lambda)d\lambda \tag{S5}$$

$$Y = k\int_{380nm}^{780nm} S(\lambda)R(\lambda)\bar{y}(\lambda)d\lambda \tag{S6}$$

$$Z = k\int_{380nm}^{780nm} S(\lambda)R(\lambda)\bar{z}(\lambda)d\lambda \tag{S7}$$

$$k = 100/\int_{380nm}^{780nm} S(\lambda)\bar{y}(\lambda)d\lambda \tag{S8}$$

The nonlinear response of the camera can be corrected by a third-order equation, and the nonlinear response correction variable is defined as $V_{Non\text{-}linear}$.

$$V_{Non-linear} = [X^3\ Y^3\ Z^3\ X^2\ Y^2\ Y^2\ X\ Y\ Z\ 1]^T \tag{S9}$$

In the dark current part of the camera, the dark current is usually a fixed value and does not change with the amount of incoming light, so a constant is given as the contribution of the dark current, and the dark current correction variable is defined as V_{Dark}.

$$V_{Dark} = [a] \tag{S10}$$

Finally, VColor is used as the base, and multiplied by the nonlinear response correction of $V_{Non\text{-}linear}$, and the result is standardized within the third order to avoid excessive correction, and finally V_{Dark} is added to obtain the variable matrix V.

$$V_{Color} = [XYZ\ XY\ XZ\ YZ\ X\ Y\ Z]^T \tag{S11}$$

$$V = [X^3\ Y^3\ Z^3\ X^2Y\ X^2Z\ Y^2Z\ XY^2\ XZ^2\ YZ^2\ XYZ\ X^2\ Y^2\ Y^2\ XY\ XZ\ YZ\ X\ Y\ Z\ a]^T \tag{S12}$$

Before using CIE DE2000 to calculate color difference, $XYZ_{Correct}$ and $XYZ_{Spectrum}$ must be converted from XYZ color space to lab color space. The conversion formula is as follows:

$$\begin{aligned} L^* &= 116f\left(\frac{Y}{Y_n}\right) - 16 \\ a^* &= 500\left[f\left(\frac{X}{X_n}\right) - f\left(\frac{Y}{Y_n}\right)\right] \\ b^* &= 200\left[f\left(\frac{Y}{Y_n}\right) - f\left(\frac{Z}{Z_n}\right)\right] \end{aligned} \tag{S13}$$

$$f(n) = \begin{cases} n^{\frac{1}{3}},\ n > 0.008856 \\ 7.787n + 0.137931, otherwise \end{cases} \tag{S14}$$

2. Training Curves

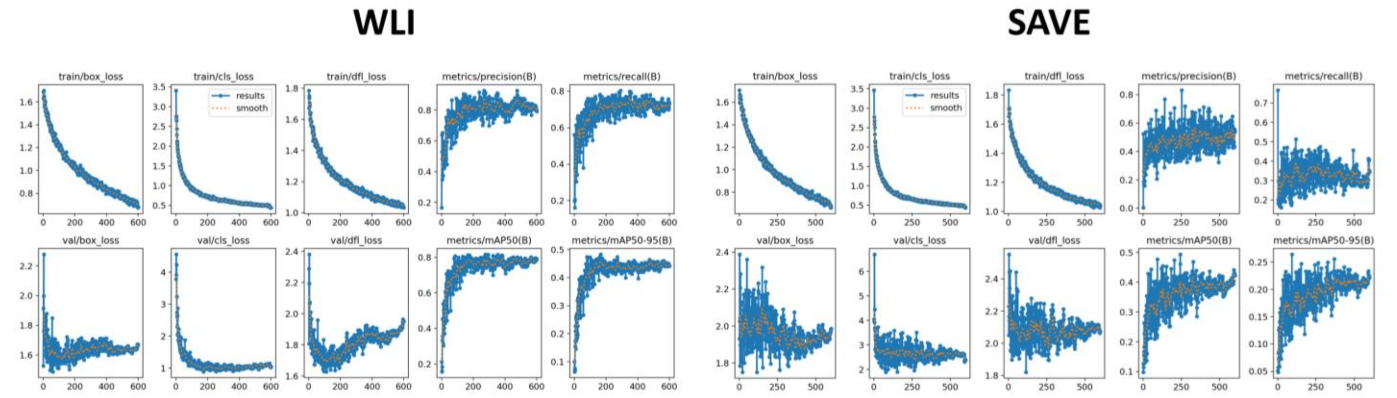

Figure S 1. Training and Loss curves of WLI and SAVE model in YOLOv5.

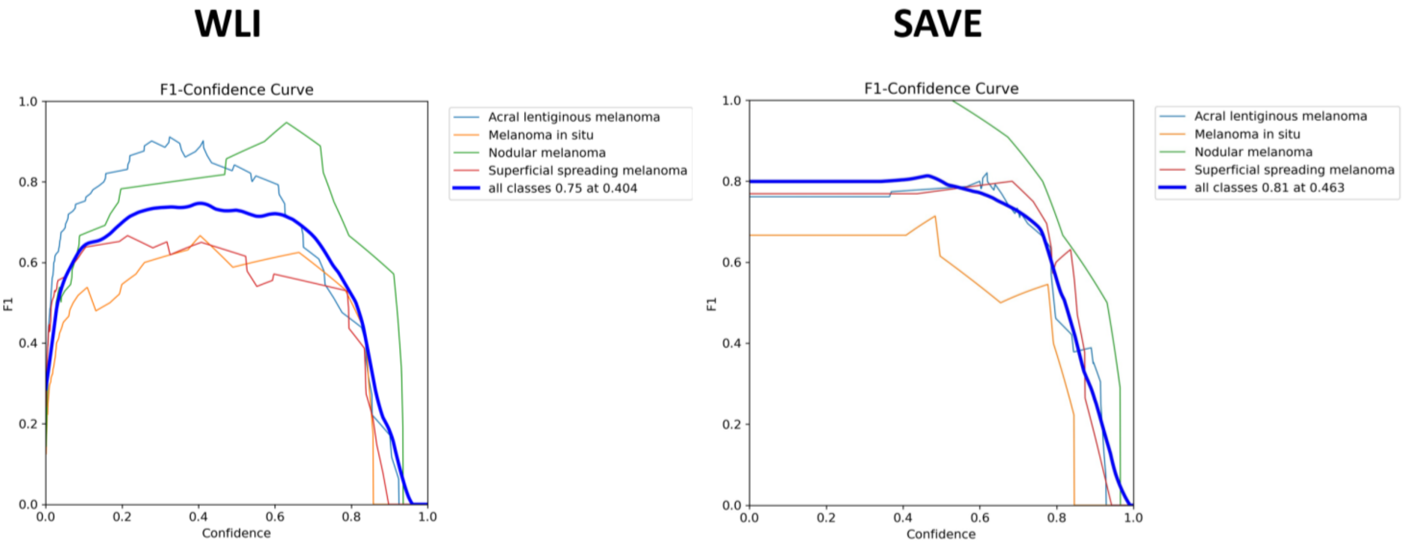

Figure S 2. F1-Confidence Curve of WLI and SAVE in YOLOv5

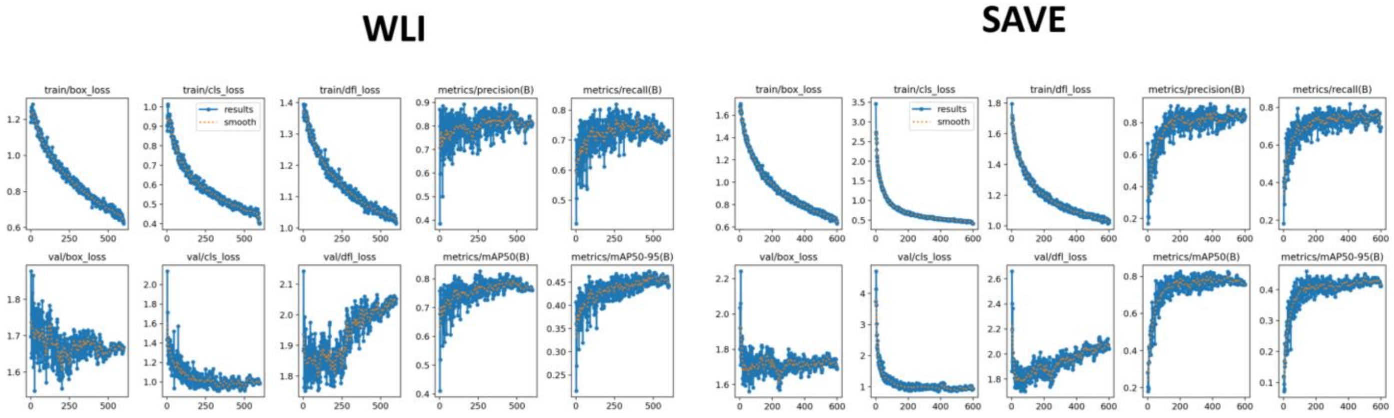

Figure S 3. Training and Loss curves of WLI and SAVE model in YOLOv8.

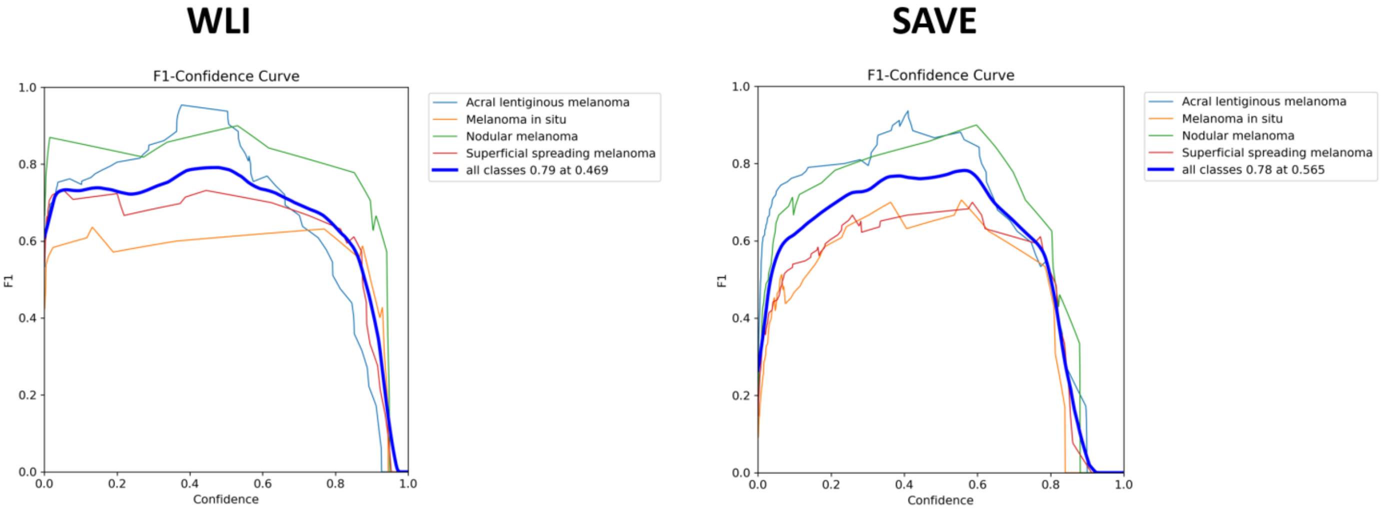

Figure S 4. F1-Confidence Curve of WLI and SAVE in YOLOv8.

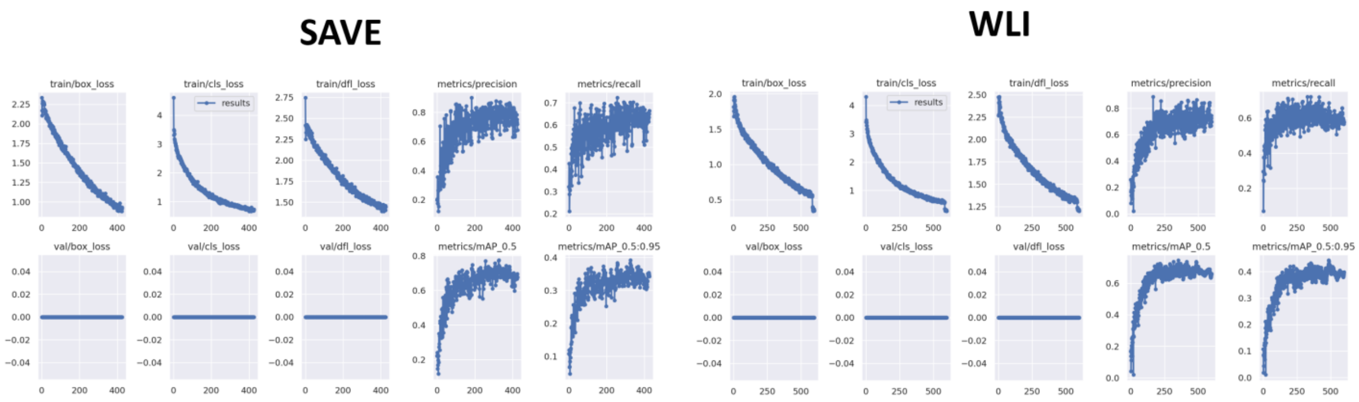

Figure S 5. Training and Loss curves of WLI and SAVE model in YOLOv9.

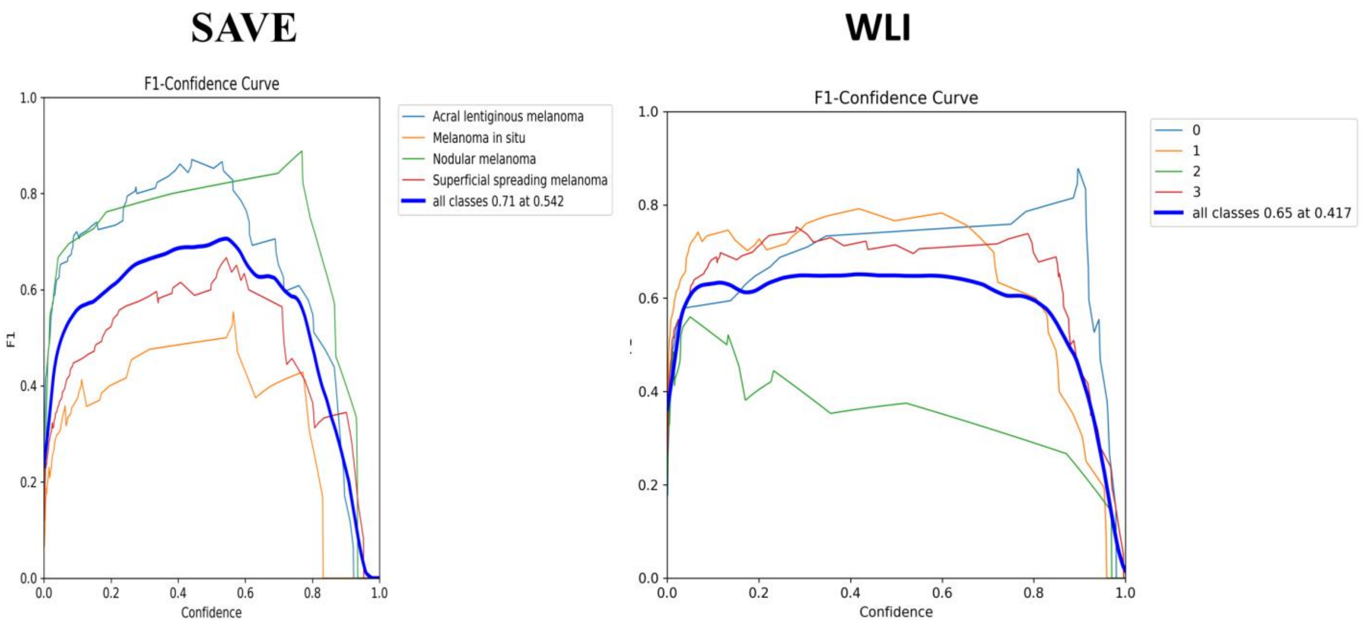

Figure S 6. F1-Confidence Curve of WLI and SAVE in YOLOv9.

3. Confusion Matrix

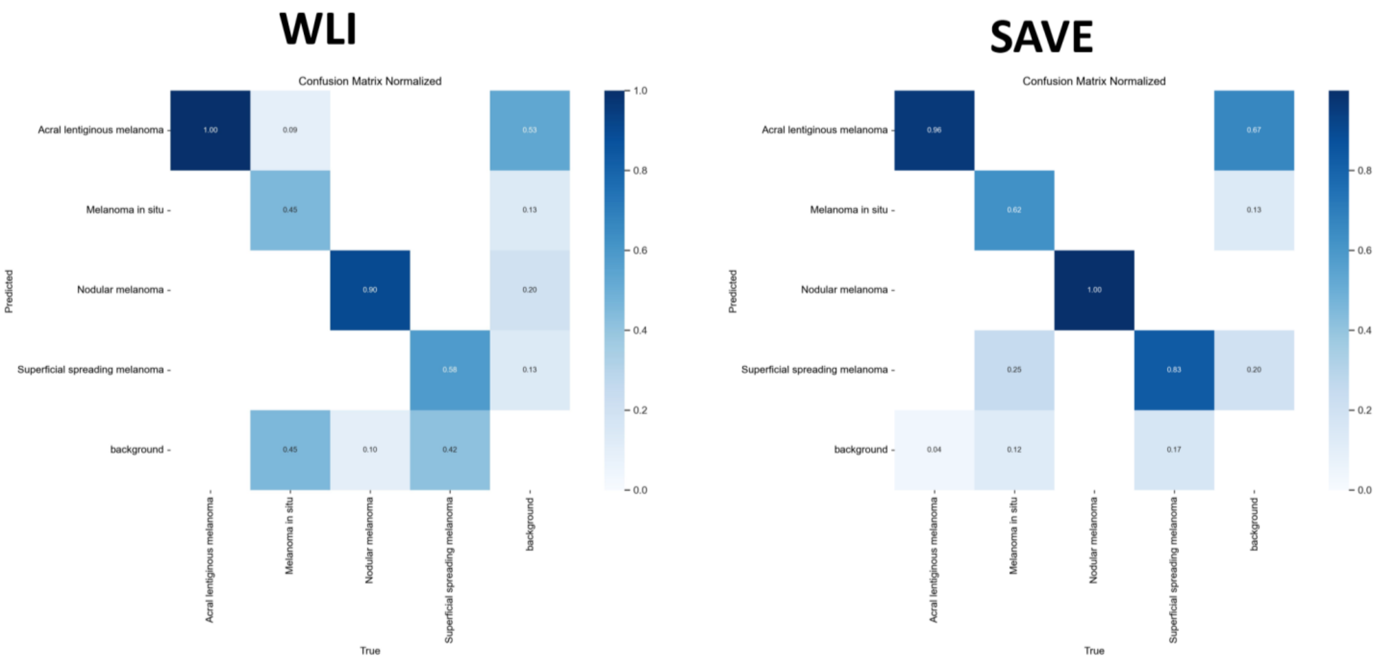

Figure S 7. Comparision between WLI and SAVE when using YOLOv5 in terms of %.

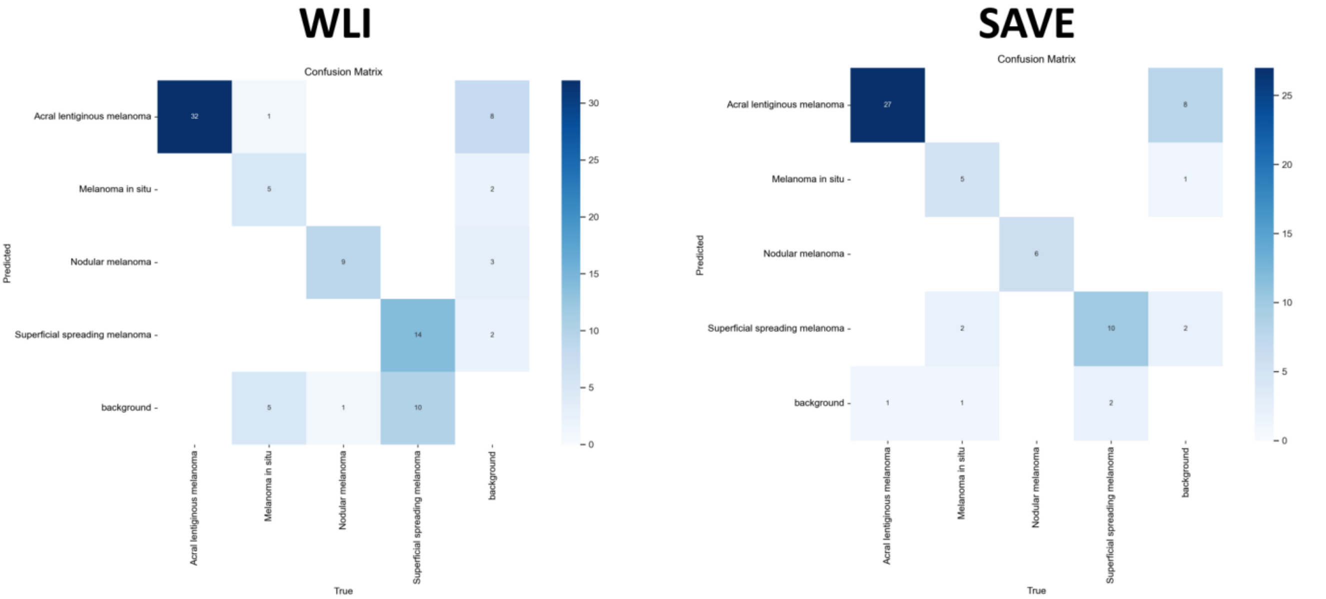

Figure S 8. Comparision between WLI and SAVE when using YOLOv5 in terms of quantitative numbers.

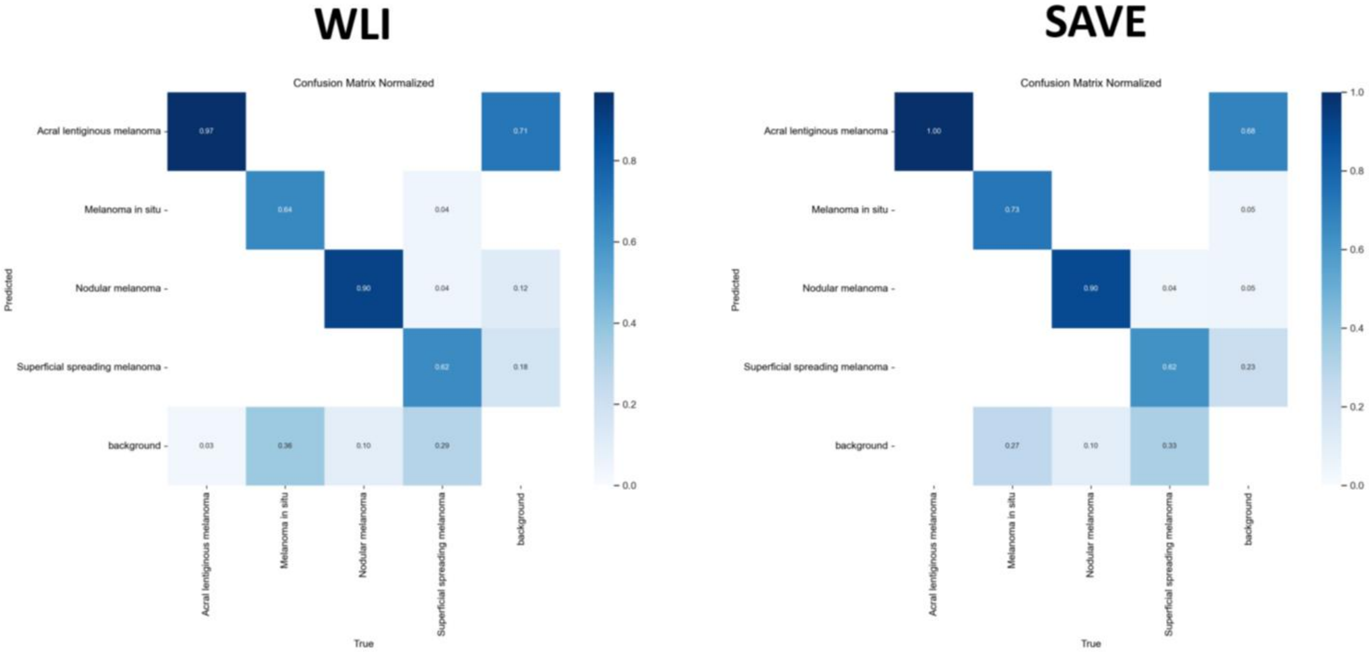

Figure S 9. Comparision between WLI and SAVE when using YOLOv8 in terms of %.

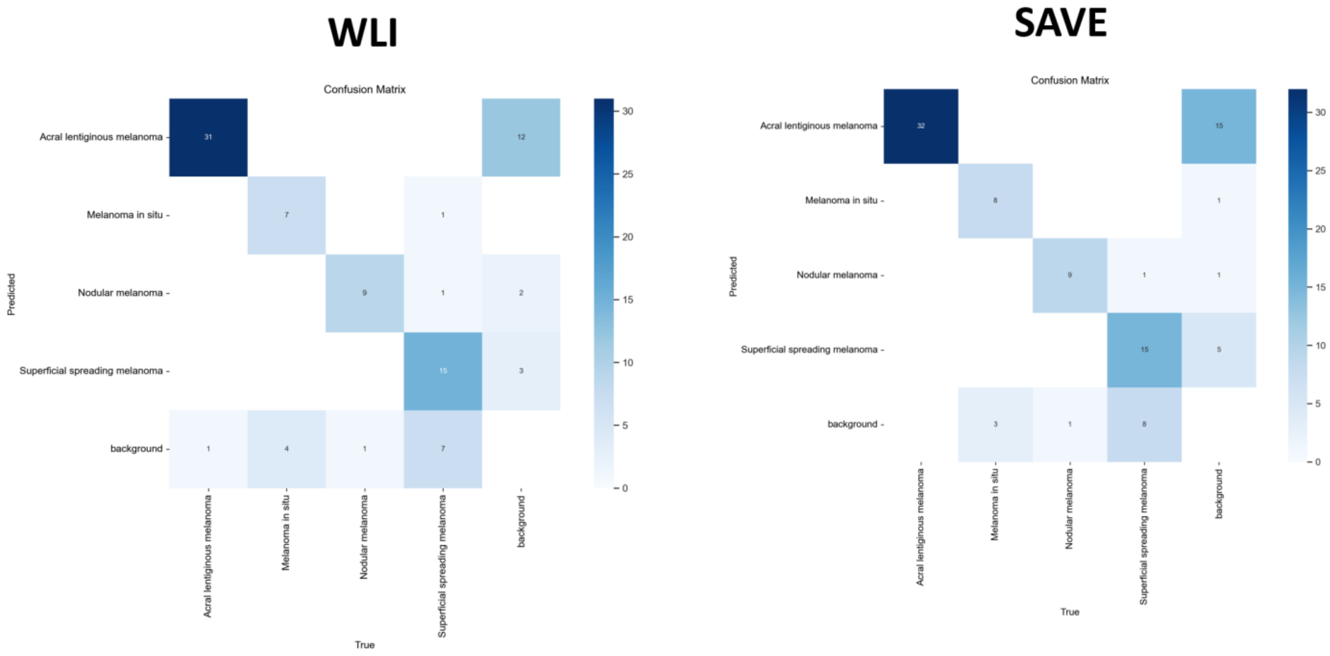

Figure S 10. Comparision between WLI and SAVE when using YOLOv8 in terms of quantitative numbers.

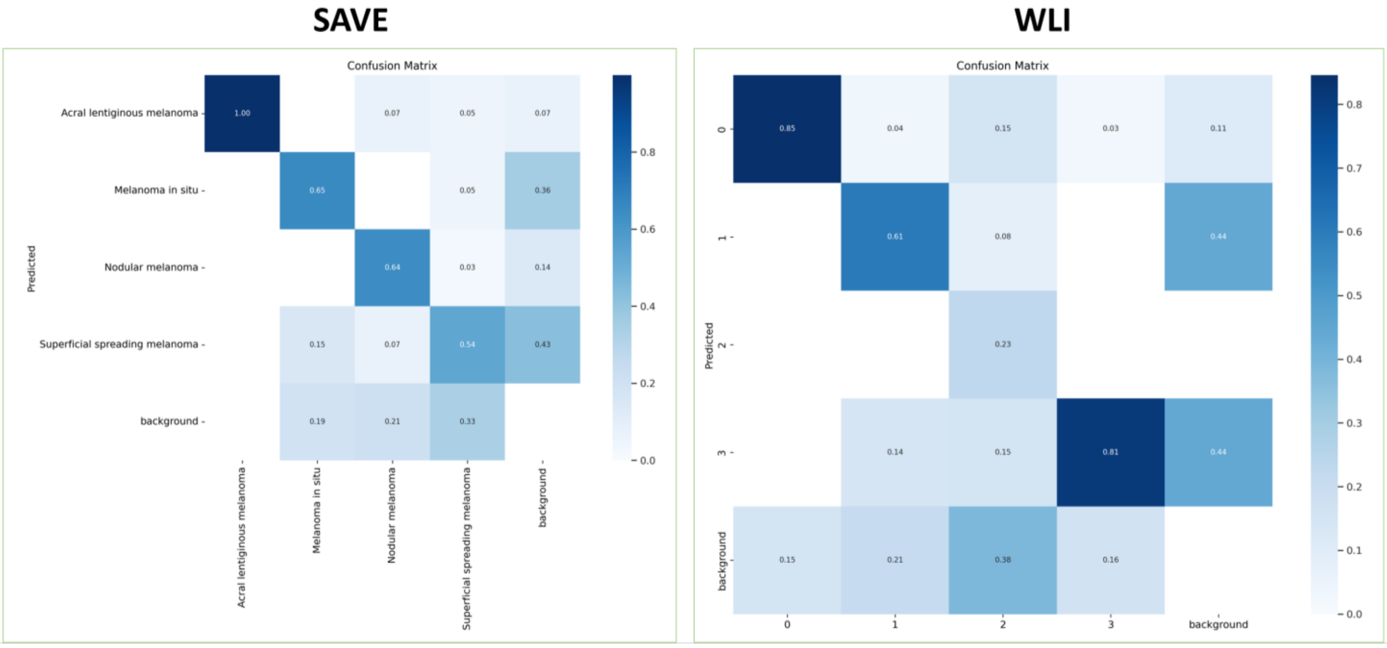

Figure S 11. Training and Loss curves of WLI and SAVE model in YOLOv9.

Predictions

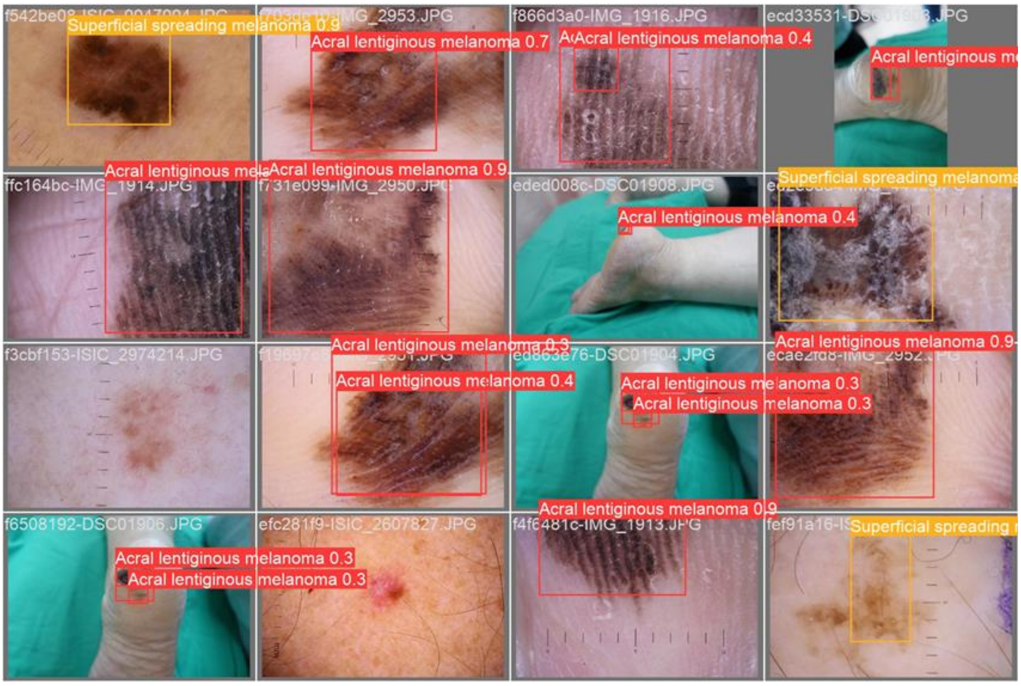

Figure S 12. Prediction results of in the RGB model.

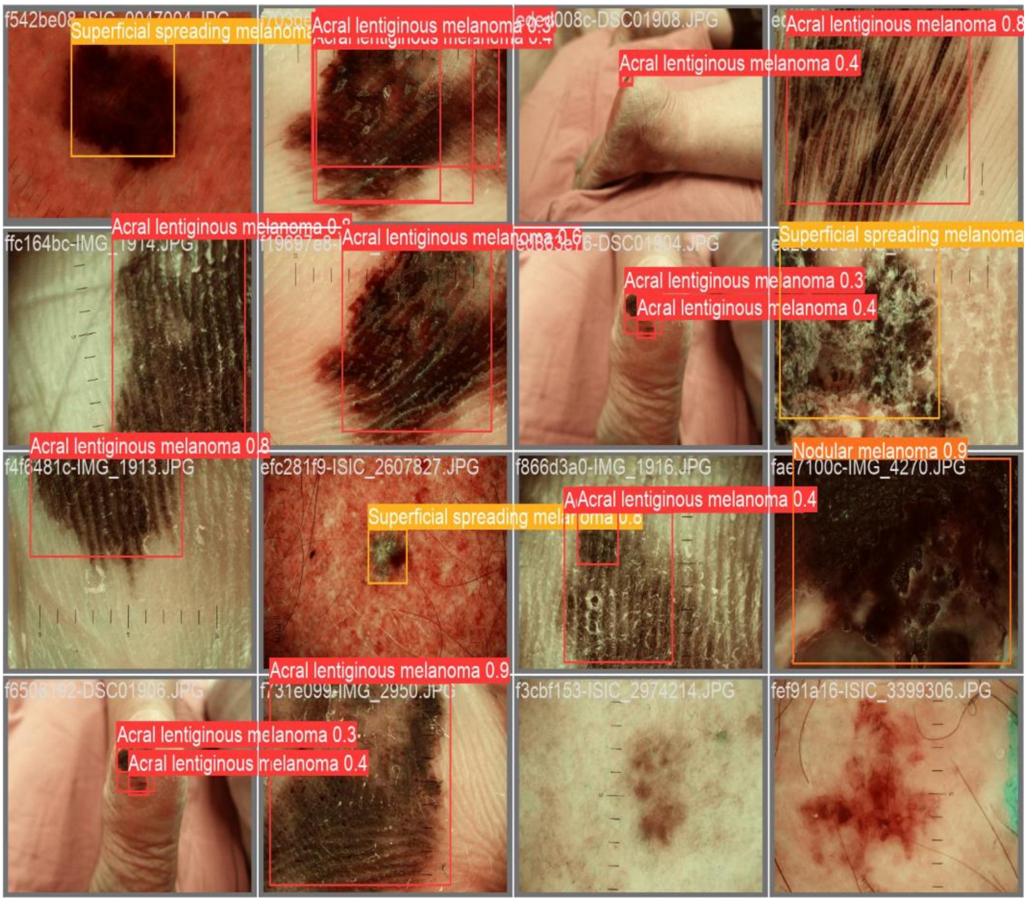

Figure S 13. Prediction results in SAVE

Dataset

Images	ALM	NM	M in Situ	SSM
**Close-up images**	231	61	21	35
**Dermoscopy images**	12	16	20	34
**Clinical images**	102	26	136	184

Disclaimer/Publisher's Note: The statements, opinions and data contained in all publications are solely those of the individual author(s) and contributor(s) and not of MDPI and/or the editor(s). MDPI and/or the editor(s) disclaim responsibility for any injury to people or property resulting from any ideas, methods, instructions or products referred to in the content.
